# A systematic review of health economic evaluations of proton beam therapy for adult cancer: Appraising methodology and quality

**DOI:** 10.1016/j.ctro.2019.10.007

**Published:** 2019-10-31

**Authors:** David A. Jones, Joel Smith, Xue W. Mei, Maria A. Hawkins, Tim Maughan, Frank van den Heuvel, Thomas Mee, Karen Kirkby, Norman Kirkby, Alastair Gray

**Affiliations:** a Health Economics Research Centre, Nuffield Department of Population Health, University of Oxford, UK; b NIHR Oxford Biomedical Research Centre, Oxford University Hospitals NHS Foundation Trust, John Radcliffe Hospital, Oxford, UK; c CRUK/MRC Oxford Institute for Radiation Oncology, Oxford, UK; d Department of Haematology/Oncology, Oxford University Hospitals NHS Foundation Trust, Oxford, UK; e Division of Cancer Sciences, School of Medical Sciences, Faculty of Biology, Medicine and Health, University of Manchester, Manchester, UK

**Keywords:** Health economic evaluation, Cost-utility analysis, Proton beam therapy, Quality evaluation

## Abstract

**Background and purpose:**

With high treatment costs and limited capacity, decisions on which adult patients to treat with proton beam therapy (PBT) must be based on the relative value compared to the current standard of care. Cost-utility analyses (CUAs) are the gold-standard method for doing this. We aimed to appraise the methodology and quality of CUAs in this area.

**Materials and Methods:**

We performed a systematic review of the literature to identify CUA studies of PBT in adult disease using MEDLINE, EMBASE, EconLIT, NHS Economic Evaluation Database (NHS EED), Web of Science, and the Tufts Medical Center Cost-Effectiveness Analysis Registry from 1st January 2010 up to 6th June 2018. General characteristics, information relating to modelling approaches, and methodological quality were extracted and synthesized narratively.

**Results:**

Seven PBT CUA studies in adult disease were identified. Without randomised controlled trials to inform the comparative effectiveness of PBT, studies used either results from one-armed studies, or dose-response models derived from radiobiological and epidemiological studies of PBT. Costing methods varied widely. The assessment of model quality highlighted a lack of transparency in the identification of model parameters, and absence of external validation of model outcomes. Furthermore, appropriate assessment of uncertainty was often deficient.

**Conclusion:**

In order to foster credibility, future CUA studies must be more systematic in their approach to evidence synthesis and expansive in their consideration of uncertainties in light of the lack of clinical evidence.

## Highlights

• The cost-effectiveness of PBT for adult cancers is subject to considerable uncertainty.
• Cost-utility analysis is the gold standard for assessing value, but quality matters.
• Our review found studies lacked external validation of model outcomes.
• When assessed against standard checklists, studies fell short.
• Improving transparency and validation will improve credibility of results.

## 1 Introduction

There is growing interest in the use of proton beam therapy (PBT) for the treatment of cancer. Unlike traditional photon based radiotherapy, protons release most of their dose at the end of their range, limiting proximal and distal irradiation. PBT therefore has the potential to reduce unwanted irradiation of normal tissues, enabling higher treatment dose for better tumour control or greater normal tissue sparing to reduce treatment-related toxicities [1].

The costs of delivering PBT are significant, with up-front capital expenditure far greater than that of a photon unit [2], [3]. Although there has been steady growth in the number of centres over the past decade, with more than 70 now operational world-wide and another 40 under construction[4], the availability of treatment remains limited. In England, for instance, the Department of Health’s recent £250 million investment in two high-energy centres creates capacity corresponding to just 1% of yearly national radiotherapy episodes [5].

With paediatric and skull base tumours already established indications for commissioning and reimbursement, uncertainty focuses on the use of remaining capacity for adult cancers, where comparative dose planning studies suggest benefit but little comparative clinical evidence as yet exists [1].

Given the limited capacity and higher costs, decisions on which adult patients to treat or evaluate in clinical trials should be based on comparisons of value against current best practice. This is typically performed through health economic evaluation (HEE). The gold standard for this is cost-utility analysis (CUA), involving the comparison of costs and effects of competing interventions with the latter expressed in quality-adjusted life years (QALYs) gained.

A number of reviews have periodically looked at the CUA literature for PBT, highlighting results and discussing important limitations due to the lack of prospectively collected data [6], [7], [8], [9], [10]. However, there has been limited focus on systematically appraising the modelling techniques used to deal with the lack of prospective data, and the quality of these models has not been systematically assessed through formal comparison to good practice guidelines [11]. This review aims to remedy these shortcomings.

## 2 Materials and methods

### 2.1 Search

A systematic search of the literature following the PRISMA guidelines [12] was conducted using the electronic databases MEDLINE, EMBASE, EconLIT, NHS Economic Evaluation Database (NHS EED), Web of Science, and the Tufts Medical Center Cost-Effectiveness Analysis Registry. Each database was searched from 1st January 2010 up to 6th June 2018, limited to the English language. The earlier time cut-off was chosen to minimise overlap with previous reviews and to focus the search on the period when the technology had become relatively more mature. Search strategies for each database are given in the Supplementary Material.

Studies met the inclusion criteria if they consisted of a full CUA, defined as a HEE where results are expressed in terms of cost per QALY gained, comparing PBT with any comparators. With paediatric disease already commonly indicated for treatment, we restricted our review to adult disease where greater decision uncertainty lies. There were no restrictions on tumour site. Conference abstracts were not eligible for inclusion. The articles identified in the search were filtered for duplicates, before titles and abstracts were screened against the inclusion criteria. Potentially relevant studies underwent a full text review.

### 2.2 Data extraction and synthesis

From each study we extracted data on their general characteristics, such as year of publication, country setting, population(s) assessed, interventions assessed, perspective, and the main results including the reported incremental cost-effectiveness ratio (ICER) of the intervention(s) using the original price year and currency. Further findings from sensitivity analyses were also extracted. These included results of probabilistic sensitivity analysis (PSA), in which model parameter values are randomly sampled from corresponding probability distributions with the model run repeatedly over the sampled parameters to create a distribution of results. The probability of being cost-effective can then be calculated for a given willingness-to-pay threshold. We also included results of any value of information analysis, which quantifies the potential gain from reducing uncertainty through further data collection.

Characteristics of each decision analytic model were also extracted, including model type, health states representing the natural history of disease, structural assumptions, time horizon, cycle length, and discount rates. Finally, we extracted the key methodological approaches in each study regarding efficacy and costs of PBT, including cited sources of information. The extracted data were presented in tables alongside narrative synthesis.

### 2.3 Assessment of study quality

A formal assessment of the quality of the studies was performed using the Philips checklist, a widely used framework for the assessment of decision analytical model-based cost-effectiveness studies [11]. The Philips checklist assesses the reporting quality across a total of 58 items. Items were answered ‘Yes’, ‘Unclear’, ‘Not Applicable’ or ‘No’. A second reviewer independently performed the checklist for a sample of the identified studies and response concordance was assessed.

## 3 Results

### 3.1 Search results

**Fig. 1:**
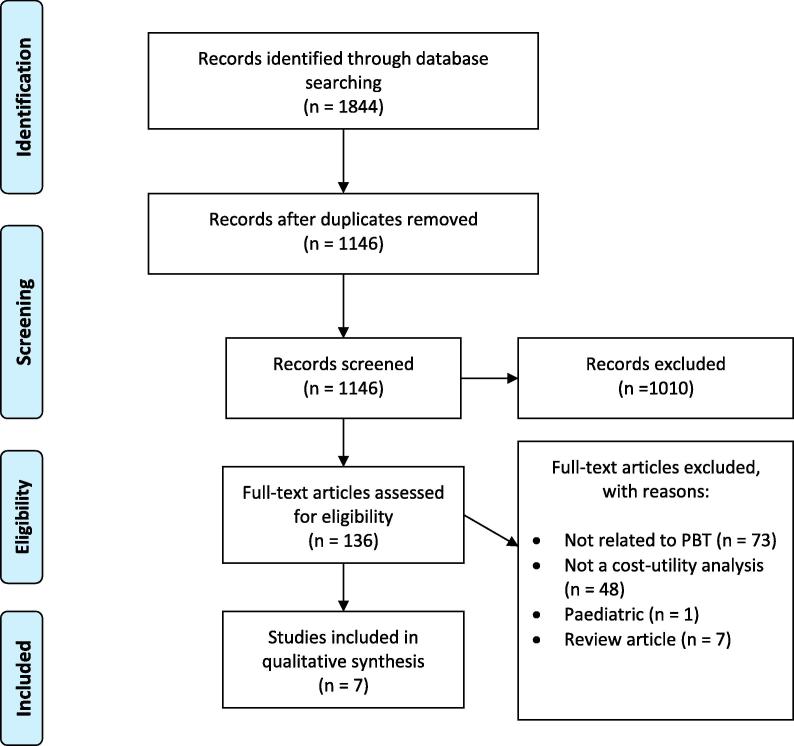
Preferred Reporting Items for Systematic Reviews and Meta-Analyses (PRISMA) diagram of study identification.

The initial search of electronic databases found 1844 articles for review. Following removal of duplicates, full-text screening and assessment of eligibility, seven papers were identified for inclusion; two for head and neck cancer (HNC) (Ramaeker et al. [13] and Sher et al. [14]), and one each for breast (Mailhot Vega et al. [15]), eye (Moriaty et al. [16]), liver (Leung et al. [17]), lung (Grutters et al. [18]), and prostate cancer (Parthan et al. [19]). A flow chart of the study selection process is shown in Fig. 1.

General characteristics of the included studies are provided in Table 1. Results from four of the identified papers suggest treatment of adult patients using PBT is cost-effective. Briefly, Leung et al. [17] found PBT to be highly cost-effective compared to SBRT for the treatment of inoperable hepatocellular carcinoma. In sensitivity analysis using the most up-to-date clinical data, Grutters et al. [18] found PBT to have the highest probability of being the most cost-effective treatment in PSA. Finally, both Ramaekers et al. [13] and Mailhot Vega [15] et al. found suggestion of cost-effectiveness at an individual patient level, dependent on predicted doses to organs at risk, and therefore subsequent risk of treatment related sequelae. These results should, however, be taken in the context of the methodological approaches used to estimate PBT effectiveness and costs, model characteristic and model quality, as outlined hereafter.

**Table 1:** General characteristics of included studies.

Study and year	Country	Cancer type	Interventions assessed	Stated Perspective	Reported main result	Other results
Grutters et al 2010	The Netherlands	Inoperable stage I non-small cell lung cancer	PBT, carbon-ion therapy, CRT, and SBRT	Dutch health Care perspective	PBT and CRT dominated by carbon-ion therapy and SBRT ICER for carbon-ion versus SBRT: €67,257	In sensitivity analysis, using evidence from studies only published after 2004: CRT dominated by carbon-ion and SBRT; ICER for carbon-ion versus SBRT, €36,017; ICER for PBT versus carbon-ion, €81,479 Value of information analysis of base case at €80,000 willingness-to-pay threshold: Population EVPI, €22 million; Parameter group most valuable for further research, Effectiveness of carbon-ion therapy
Parthan et al 2012	USA	Localized prostate cancer	PBT, IMRT, and SBRT	Health care payer and societal	PBT and IMRT dominated by SBRT in both perspectives	–
Ramaekers et al 2013	The Netherlands	Locally advanced (stage 3–4) head and neck cancer	PBT for all patient, IMRT for all patients, and PBT if efficient	Dutch health Care perspective	ICER for PBT if efficient versus IMRT for all: €60,278 ICER for PBT for all versus IMPT if efficient: €127,946	In sensitivity analysis, the relaxed assumption of equal disease progression, taking estimates from a synthesis of clinical studies, caused PBT to be dominated by IMRT for all patients Value of information analysis of base case at a €80,000 willingness-to-pay threshold: Population EVPI, €2.4 million Parameters most valuable for further research: Utility scores after xerostomia, NTCP models for dysphagia and xerostomia
Moriaty et al 2015	USA	Intraocular melanoma	PBT, enucleation, and plaque brachytherapy	Provider perspective	ICER for PBT versus enucleation: $106,100 ICER for plaque brachytherapy versus enucleation: $77,500 ICER for PBT versus plaque brachytherapy not reported	–
Mailhot Vega et al 2016	USA	Breast cancer	PBT and photon radiotherapy	Societal perspective	In base case analysis with $50,000 threshold: Women with no CRFs: PBT not cost-effective for all ages and for all photon MHD tested (up to 10 Gy) Women with CRFs: PBT cost-effective for 50- and 60-year-old women with MHD of 9 Gy and 10 Gy respectively In base case analysis with $100,000 threshold: Women with no CRFs: PBT cost-effective for 40- and 50-year-old women with MHD of 10 Gy and 9 Gy respectively Women with CRFs: PBT cost-effective for 40-, 50- and 60-year-old women with MHD of 6 Gy, 5 Gy and 6 Gy respectively.	In PSA analysis with $50,000 threshold: Women with no CRFs - PBT not cost-effective for all ages and for all photon MHD tested (up to 10 Gy) Women with CRFs - PBT cost-effective for 40, 50- and 60-year-old women with MHD of 9 Gy, 7 Gy and 8 Gy respectively In PSA analysis with $100,000 threshold: Women with no CRFs - PBT cost-effective for 40, 50- and 60-year-old women with MHD of 9 Gy, 7 Gy and 9 Gy respectively Women with CRFs - PBT cost-effective for 40-, 50- and 60-year-old women with MHD of 5 Gy, 4 Gy and 5 Gy respectively.
Leung et al 2017	Taiwan	Inoperable advanced hepatocellular carcinoma (large tumours)	PBT and SBRT	Single payer healthcare system	ICER for PBT versus SBRT: NT$ 213,354 (equivalent to US $14,180 in 2016 prices)	–
Sher et al 2018	USA	Oropharyngeal squamous cell carcinoma	PBT and IMRT	Payer perspective and societal perspective	HPV-positive patients: ICERs for PBT versus IMRT: $288,000 and $390,000 in the payer and societal perspectives respectively HPV-negative patients: ICERs for PBT versus IMRT: $516,000 and $695,000 in the payer and societal perspectives respectively	In one-way sensitivity analysis, even under assumptions that strongly favoured the efficacy of PBT to reduce PEG dependence or improve long-term xerostomia, the ICERs were uniformly above $100 K Value of information analysis for 55 year old patients: EVPI at $100 K willingness-to-pay threshold, $185,000 in payer perspective and $0 for societal perspective; EVPI at $150 K willingness-to-pay, $71 million in payer perspective and $2.4 million in societal perspective Value of information analysis for 65 year old patients: EVPI negligible for the different willingness-to-pay thresholds and perspectives

*Footnote:* PBT, Proton Beam Therapy; CRT, conventional radiotherapy; SBRT, Stereotactic Body Therapy; ICER, Incremental Cost-Effectiveness Ratio; EVPI, Expected Value of Perfect Information; IMRT, Intensity Modulated Radiation Therapy; CRF, Cardiac Risk Factor; MHD, Mean Heart Dose; PSA, Probabilistic Sensitivity Analysis; PEG, Percutaneous Gastrostomy Tube.

### 3.2 Methodological approaches and sources for PBT effectiveness and costs

With randomised controlled trials to inform comparative effectiveness lacking, other approaches to estimate the effect of PBT were employed. Four studies [16], [17], [18], [19] derived efficacy estimates from single-armed trials and observational data: Grutters et al. [18] *meta*-analysed results from systematically identified single-armed studies to inform survival, disease progression, treatment-related death, and occurrence of grade 3–5 (CTCAE scoring system) pneumonitis, oesophagitis, and irreversible dyspnoea for each of the compared interventions. Similarly, Moriaty et al. [16] “pooled” results of single-armed studies to inform disease progression after treatment to local recurrent and metastatic health states, as did Parthan et al. [19] to inform probability of long-term toxicities. Leung et al. [17] derived treatment effect estimates for disease progression and incidence of severe adverse events (≥grade 3) from two single-armed phase II trials, one each for PBT [20] and SBRT [21].

An alternative approach, applying risk stratification through predictive dose–response models derived from radiobiological and epidemiological studies of photon radiotherapy outcomes, was employed by two studies [13], [15]. Ramaekers et al. [13] estimated risk of suffering xerostomia and dysphagia after intensity modulated radiotherapy (IMRT) and PBT at an individual patient-level using dosimetry data from a comparative planning study [22] of 25 patients (oropharyngeal (n = 21) and hypopharyngeal (n = 4)) linked to normal tissue complication probability (NTCP) models. Both NTCP modelling studies [23], [24] used logistic regression to estimate the association of patient baseline characteristics and dose distributions to organs at risk from dose-volume histograms (DVH) on the probability of developing post-treatment toxicity in prospective cohorts. Mailhot Vega et al. [15] estimated bounds for cost-effective treatment depending again on dosimetry and baseline patient characteristics. Fixing mean heart dose from PBT at 0.5 Gy, the authors varied photon mean heart dose and used a population-based case–control study [25] of the association between mean heart dose and risk of ischemic heart disease to establish photon doses at which the increased risk under photon based therapy would make PBT cost-effective.

Finally, in their base-case analysis Sher et al. [14] informed fixed reductions in odds of developing toxicities from the results of studies linking comparative patient treatment plans with NTCP models [26], [27], an unadjusted comparison of consecutive IMRT and PBT patient [28], and a case-matched prospective cohort study [29]. Similar to Maihot Vega et al. [15], they performed sensitivity analysis to identify a theoretical toxicity reduction threshold at which PBT would become cost-effective.

Approaches and sources used to estimate proton related treatment costs ranged across studies in relation to their jurisdiction and perspective. Both Grutters et al. [18] and Ramaekers et al. [13] conducted their analyses from a Dutch health-care perspective, making use of a previous costing analysis [3] by their research group. Capital and operational costs of constructing and running a proton facility were incorporated as well as various other assumptions on rate of use and case-mix. A similar costing approach was used in Mailhot Vega et al. [15] and Sher et al. [14] from a US societal perspective.

Sher et al. [14] also used Medicare reimbursement rates to estimate cost per treatment from a payer perspective, as did Parthan et al. [19], although garnered from another published source [30]. The latter also incorporated an estimate of the age-specific opportunity cost of lost time due to radiotherapy into a societal analysis. Medicare reimbursement rates were also used by Moriaty et al. [16] who then applied an adjustment to account for discrepancies between billed charges and actual resource use for a provider perspective. Finally, Leung et al. [17] simply assumed an insurance reimbursement package of NT$300,000 for PBT (equivalent to US $19,938 in 2016 prices).

### 3.3 Model characteristics and quality

**Fig. 2:**
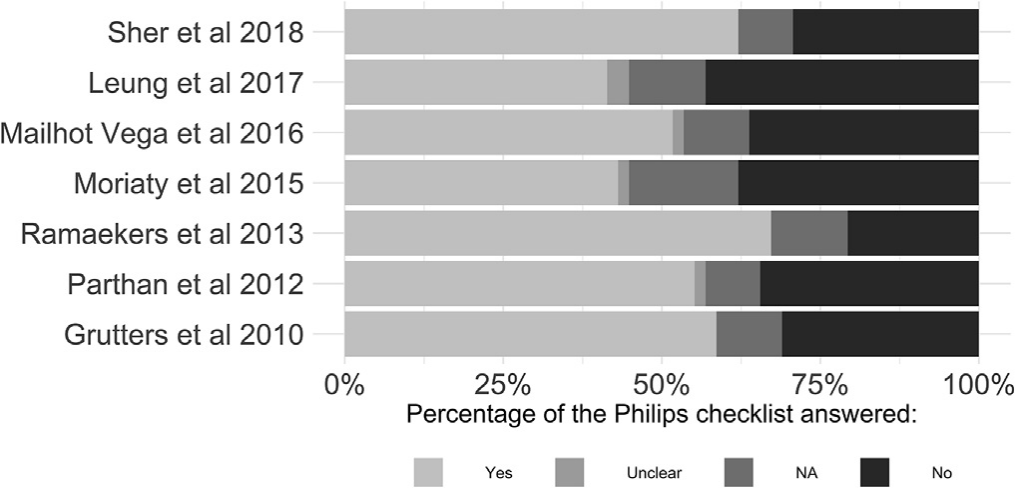
Aggregate results of the Philip’s checklist.

Also key in driving results are the characteristics and quality of the decision analytic models used. Model characteristics are summarised in Table 2 whilst aggregated results of the Philips checklist for each study are presented in Fig. 2. A second independent reviewer performed the checklist on two of the studies [16], [17], finding a high level of agreement, with concordance in 87% of total items. Below we outline key findings.

**Table 2:** Model characteristics of included studies.

Study	Decision model type	Health states (including toxicities)	Structural assumptions	PBT treatment effect assumptions	Time horizon	Cycle length	Discount rates
Grutters et al 2010	Cohort Markov model	Intermediate states representing pneumonitis (≥grade 3), oesophagitis (≥grade 3), or treatment related death in first 6 weeks of treatment. Alive without dyspnoea (≥grade 3). Alive with irreversible dyspnoea (≥grade 3). Dead	No second malignancies in model. Pneumonitis and oesophagitis only during the 6 weeks of treatment. Dyspnoea was irreversible	Overall and disease-specific survival rates, as well as the occurrence pneumonitis (≥grade 3), oesophagitis (≥grade 3), irreversible dyspnoea (≥grade 3), and grade 5 adverse events (treatment-related death) were all extracted from systematic review and *meta*-analysis	5 years	Yearly	Effects: 1.5% Costs: 4%
Parthan et al	Cohort Markov model	No long-term toxicities, GU, GI, SD, GU & GI, GU & SD, GI & SD, GU & GI & SD, Dead	Toxicities irreversible	Equal disease progression. Radiotherapy modality affected long-term toxicity probability.	Lifetime	Did not explicitly state, yearly implied	Effects: 3% Costs: 3%
Ramaekers et al 2013	Cohort Markov model	Disease free with no toxicity, Disease free with xerostomia (≥grade 2), Disease free with dysphagia and xerostomia (≥grade (≥grade 2), Disease free with dysphagia (≥grade 2), Loco-regional recurrence, Distant metastasis, and Dead	Toxicity that occurred in the first 6 months was potentially reversible. Thereafter it was irreversible. No transition between loco-regional recurrence and Distant metastasis and vice versa due to short life expectancy in the latter state	Equal disease progression. Radiotherapy modality affected outcomes through occurrence of xerostomia and or dysphagia. This was estimated via two NTCP models with dosimetric variables as inputs, estimated for each patient using comparative planning of IMPT and IMRT	Lifetime	A cycle time of 6 months was used in the first year, afterward the cycle time was 1 year	Effects: 1.5% Costs: 4%.
Moriaty et al 2015	Cohort Markov model	Post treatment, Local recurrence, Distant metastasis, Dead from disease, Dead from other causes	No acute or long term toxicities from treatment	Risk of metastatic cancer after local recurrence the same for each intervention due to lack of evidence	5 years	Yearly	Effects: 3% Costs: 3%
Mailhot Vega et al 2016	Cohort Markov model	Healthy, alive with coronary heart disease, Dead	In basecase analysis, CHD was managed purely medically. In sensitivity analysis, occurrence of percutaneous coronary intervention in either inpatient and outpatient setting was incorporated	No difference in tumour control. Proton therapy delivered a mean heart does of 0.5 Gy	Lifetime	Yearly	Effects: 3% Costs: 3%
Leung et al 2017	Cohort Markov model	Stable disease, Disease progression, Dead	Severe toxicities only incorporated as extra costs and utility decrement	Treatment effect on disease progression from clinical trial	5 years	Monthly	Effects: Did not state Costs: 3%
Sher et al 2018	Cohort Markov model	No evidence of disease (could include toxicities: PEG, dysgeusia, and xerostomia), Locoregional recurrence, Distant metastases, Dead (other, and OPC related)	No transition between loco-regional recurrence and distant metastasis and vice versa	No difference in tumour control. Reduced risk of toxicities with PBT. Odds ratio: Dysgeusia − 0.75, Xerostomia − 0.75, PEG − 0.75	Lifetime	Monthly	Effects: 3% Costs: 3%

*Footnote:* GU, Genitourinary; GI, Gastrointestinal; SD, Sexual Dysfunction; NTCP, Normal Tissue Complication Probability; PBT, Proton Beam Therapy; IMRT, Intensity Modulated Radiation Therapy; CHD, Coronary Heart Disease; PEG, Percutaneous Gastrostomy Tube.

Each of the seven studies used discrete-time cohort Markov models to simulate the natural history of disease. However, there was a general lack of explicit rationale for the disease model structure, with no references to the natural history of disease literature or other disease models. Despite this, structural assumptions were mostly transparent and justified. Just under half of the studies [16], [17], [18] used a 5-year time horizon with only one [18] justifying their choice due to a lack of clinical evidence past this horizon.

Systematic identification of disease progression parameters was performed in only two studies [13], [18], with one other conducting a literature review of publications from active research groups in the disease area [16]. These studies did not apply the same methods to other parameters such as utility values, or formally assess the quality of included data. Expert opinion was used in four studies [13], [17], [18], [19] to estimate model parameter value, but the methods used for elicitation were not transparent.

As previously mentioned, four studies [16], [17], [18], [19] derived efficacy estimates from single-armed trials or observational data. Grutters et al. [18] *meta*-analysed results from systematically identified single-armed studies, Parthan et al. [19] and Moriaty et al. [16] pooled results of single-armed studies without specifying the method, and Leung et al. [17] used treatment effect estimates from two single-armed phase II trials. In none of these evaluations were methods used to adjust for the use of non-comparative efficacy data, introducing potential confounding caused by patient selection bias.

Utilities were found to be poorly reported in three studies. Moriaty et al. [16] applied a slight utility decrement to the post treatment state after proton beam therapy but gave no clear rationale as to why, while Mailhot Vega et al. [15] did not state the utility derived from being in a healthy state. Leung et al. [17] applied a reduction in utility according to the incidence rate of severe adverse event (≥grade 3) reported in the treatment effect trials, but provided no numeraire or reference for the weight of reduction.

Whilst all studies considered some form of uncertainty, none addressed all, and no justification was given for their absence. Parameter uncertainty was assessed using either one-way sensitivity analysis [14], [16], [17], [19], in which one parameter is varied whilst the others are kept constant, and/or probabilistic sensitivity analysis (PSA) [13], [14], [15], [17], [18], [19]. PSA was not always conducted thoroughly. In Mailhot Vega et al. [15] only three parameters were given probability distributions, Sher et al. [14] did not state the parametric form of the distributions for most parameters, and Leung et al. [17] did not base the scale and shape of the distributions on actual data, rather ranging all parameters by ±30% of the base estimate. Heterogeneity in results was only considered in three studies [13], [14], [15], and only two [13], [15] and one [18] studies considered structural and methodological uncertainty respectively.

Finally, in regards to external consistency, calibration of natural history of disease outputs against independent data was performed in only one study [14], with appropriate justification of differences. Although only one study was identified for most disease sites, no studies made any reference to other cost-effectiveness models in their disease area, to which absolute outcomes for the comparator could be compared. In HNC, where two models were developed, Sher et al. [14] made no reference to the earlier work of Ramaekers et al. [13], and therefore did not explore the reasons for differences in their findings.

## 4 Discussion

Although several reviews of the PBT HEE literature have been performed, none have looked in-depth at the methodological approaches to modelling costs and effects, nor assessed the quality of the models. Even within the wider radiation oncology literature, reviews of HEE study quality have been performed infrequently [31], [32], [33]. Such reviews are important, as any inference drawn from modelling studies must be considered in light of the rigour of the analysis.

Our appraisal of model quality using the Philips checklist found limitations in most of the seven identified studies in terms of their transparency (clarity in the description and assumptions of the model and identification of model inputs) and validity (how well the model reflects reality). Transparency was hindered by a lack of systematic methods or even an explanation for the identification of model parameters, such as transition probabilities, health state costs, and utilities. Although not a guarantor of a model’s internal validity, transparency of model input choices is a key requirement of credibility, allowing decision makers to accurately assess the merits of the study and any biases introduced through selective choice of inputs [34]. External validation of the model outputs was also lacking. Again, although not a guarantor of a model’s external validity, the comparison of model outputs against independent data and cross-comparison with results from other CUA model outputs provide credibility that the model accurately reflects reality. Similar findings for transparency were noted in two other reviews of HEE study quality in the wider radiation oncology literature, suggesting this is not a problem exclusive to PBT, whilst the lack of external validation is a perennial problem within HEE [31], [32], [35] Through greater transparency and assessment of external consistency, future CUAs of PBT will increase confidence in the reliability of their analysis, and validity of their findings.

A specific problem posed in the HEE of PBT is the well-documented lack of prospectively collected comparative data, especially from randomised controlled trials [6], [7], [8], [9], [10]. Estimating the costs of delivering PBT, and radiotherapy in general, is notably complex [36]. Although laudable efforts to improve and standardise costing methodology are ongoing, our review found large variations [2], [37]. Our review also highlights the divergent methodological approaches to estimate PBT effectiveness taken by these studies, each with their own issues. Treatment effect estimates based on single-armed trials are likely to introduce confounding due to patient selection bias, whilst the use of NTCP models may not be generalisable over time or to the biological response induced by proton irradiation [38], [39], [40], [41]. Given these limitations and variation, sensitivity analysis and adequate expression of uncertainty should have been a key feature of the studies, but our appraisal of model quality often found deficiencies. Good practice assessment of total parameter uncertainty through PSA was only performed and reported suitably in two studies [13], [18]. This is surprising considering the inherent strength of model-based CUA analysis for quantifying uncertainty around any point estimates and the problem being addressed [42].

Furthermore, well conducted quantification of parameter uncertainty though PSA can be harnessed to infer the value and prioritisation of future data collection through value of information analysis. Uncertainty in results leads to the risk of making a suboptimal treatment decision, incurring a loss in health and healthcare resources compared to the optimal choice. Reducing decision uncertainty through further data collection therefore has quantifiable value by increasing the probability of making the optimal treatment decision. Less than half of our identified studies [13], [14], [18] estimated the expected value of perfect information (EVPI), a measure of the ceiling value of reducing all uncertainty through future research. And only two [13], [18] estimated the expected value of perfect parameter information (EVPPI), in which a ceiling value can be attributed to each source of uncertainty [43]. Value of information analysis can be yet further extended through the expected value of sample information (EVSI) and the expected net benefit of sampling (ENBS) to determine the benefit of reducing uncertainty through a future study, taking into account the cost of research [44], [45]. Recent methodological advances have significantly reduced the computational burden of such analyses, whilst software packages have made computation far more accessible [46], [47], [48], [49], [50], [51]. With many countries now running or establishing proton therapy centres, such analyses may help optimise the appropriate allocation of limited research funding and treatment capacity.

A limitation of our review is its dependence on what was reported within the identified papers and any supplements: we had insufficient time or resource to contact individual authors to seek clarification. The Philips checklist has an element of subjectivity arising from individual interpretation of checklist items, and had resources permitted, secondary assessment would have been performed on all papers.

In conclusion, our review indicates that lack of transparency or external validation were key areas for improvement in future CUAs. The full reporting of uncertainty should be undertaken, ideally through PSA, which will also allow for the application of more advanced HEE methods to inform data collection and future research. The fast pace of developments in PBT will hopefully lead to a greater frequency of CUAs, and our review will hopefully provide direction on where their quality should be improved.

## Declaration of Competing Interest

The authors declare that they have no known competing financial interests or personal relationships that could have appeared to influence the work reported in this paper
