# Supplementary material for: A systematic review of health economic evaluations of proton beam therapy for adult cancer: Appraising methodology and quality
Source: Clin Transl Radiat Oncol. 2019 Oct 31;20:19–26. doi: 10.1016/j.ctro.2019.10.007 (PMC6854069; doi:10.1016/j.ctro.2019.10.007)
Supplement: Supplementary Data 1 [file mmc1.docx]

**Supplementary material**

Search strategies for each of the databases searched

| **Medline** | Searches |
| --- | --- |
| 1 | Proton Therapy/ |
| 2 | ((proton beam or particle) adj3 (therap* or treatment)).ti,ab. |
| 3 | ((proton? or particle) adj3 (radiotherap* or radiation therap* or radiation treatment)).ti,ab. |
| 4 | exp Stereotaxic Techniques/ |
| 5 | (stereota* adj3 (radiotherap* or radiation therap* or radiation treatment)).ti,ab. |
| 6 | (sbrt or sabr).ti,ab. |
| 7 | Radiotherapy, Intensity-Modulated/ |
| 8 | (intensity modulated adj3 (radiotherap* or radiation therap* or radiation treatment)).ti,ab. |
| 9 | (volumetric modulated arc adj3 (radiotherap* or radiation therap* or radiation treatment)).ti,ab. |
| 10 | (imrt or vmat).ti,ab. |
| 11 | linac.ti,ab. |
| 12 | (conformal adj3 (radiotherap* or radiation therap* or radiation treatment)).ti,ab. |
| 13 | 1 or 2 or 3 or 4 or 5 or 6 or 7 or 8 or 9 or 10 or 11 or 12 |
| 14 | economics/ or exp economics, hospital/ or exp economics, medical/ |
| 15 | "Value of Life"/ |
| 16 | exp health care costs/ |
| 17 | economic evaluation*.ti,ab. |
| 18 | (Cost* adj2 (Effective* or analysis* or Utility* or Benefit* or Minimi*)).ti,ab. |
| 19 | (pharmacoeconomic* or pharmaco-economic*).ti,ab. |
| 20 | economic*.ti. |
| 21 | "willingness to pay".ti,ab. |
| 22 | ((economic adj2 model*) or markov or monte carlo method).ti,ab. |
| 23 | (decision* adj (tree* or model* or analysis)).ti,ab. |
| 24 | (resource* adj (use* or utilisation)).ti,ab. |
| 25 | ((healthcare or health care or direct service or hospital or drug*) adj cost*).ti,ab. |
| 26 | 14 or 15 or 16 or 17 or 18 or 19 or 20 or 21 or 22 or 23 or 24 or 25 |
| 27 | 13 and 26 |
| 28 | limit 27 to yr="2010 -Current" |
| 29 | limit 28 to english language |

| **EMBASE** | Searches |
| --- | --- |
| 1 | *Proton Therapy/ |
| 2 | ((proton beam or particle) adj3 (therap* or treatment)).ti,ab. |
| 3 | ((proton? or particle) adj3 (radiotherap* or radiation therap* or radiation treatment)).ti,ab. |
| 4 | *stereotactic body radiation therapy/ |
| 5 | (stereota* adj3 (radiotherap* or radiation therap* or radiation treatment)).ti,ab. |
| 6 | (sbrt or sabr).ti,ab. |
| 7 | *intensity modulated radiation therapy/ or *volumetric modulated arc therapy/ |
| 8 | (intensity modulated adj3 (radiotherap* or radiation therap* or radiation treatment)).ti,ab. |
| 9 | (volumetric modulated arc adj3 (radiotherap* or radiation therap* or radiation treatment)).ti,ab. |
| 10 | (imrt or vmat).ti,ab. |
| 11 | linac.ti,ab. |
| 12 | (conformal adj3 (radiotherap* or radiation therap* or radiation treatment)).ti,ab. |
| 13 | 1 or 2 or 3 or 4 or 5 or 6 or 7 or 8 or 9 or 10 or 11 or 12 |
| 14 | health economics/ or pharmacoeconomics/ |
| 15 | exp health care costs/ |
| 16 | economics/ or economic aspect/ |
| 17 | economic evaluation*.ti,ab. |
| 18 | (Cost* adj2 (Effective* or analysis* or Utility* or Benefit* or Minimi*)).ti,ab. |
| 19 | (pharmacoeconomic* or pharmaco-economic*).ti,ab. |
| 20 | economic*.ti. |
| 21 | "willingness to pay".ti,ab. |
| 22 | ((economic adj2 model*) or markov or monte carlo method).ti,ab. |
| 23 | (decision* adj (tree* or model* or analysis)).ti,ab. |
| 24 | (resource* adj (use* or utilisation)).ti,ab. |
| 25 | ((healthcare or health care or direct service or hospital or drug*) adj cost*).ti,ab. |
| 26 | 14 or 15 or 16 or 17 or 18 or 19 or 20 or 21 or 22 or 23 or 24 or 25 |
| 27 | 13 and 26 |
| 28 | limit 27 to yr="2010 -Current" |
| 29 | limit 28 to english language |

| **EconLIT** | noft((("proton beam" or particle) N3 (therap* or treatment))) OR noft(((proton* or particle) N3 (radiotherap* or "radiation therap*" or "radiation treatment"))) OR noft((stereota* N3 (radiotherap* or "radiation therap*" or "radiation treatment"))) OR noft(sabr OR sbrt) OR noft(("intensity modulated" N3 (radiotherap* or "radiation therap*" or "radiation treatment"))) OR noft(("volumetric modulated arc" N3 (radiotherap* or "radiation therap*" or "radiation treatment"))) OR noft(imrt OR vmat OR linac) |
| --- | --- |

| **NHSEED** | Search |
| --- | --- |
| #1 | MeSH descriptor: [Proton Therapy] explode all trees |
| #2 | (("proton beam" or particle) near/3 (therap* or treatment)):ti,ab,kw or ((proton* or particle) near/3 (radiotherap* or "radiation therap*" or "radiation treatment")):ti,ab,kw (Word variations have been searched) |
| #3 | MeSH descriptor: [Stereotaxic Techniques] explode all trees |
| #4 | (stereota* near/3 (radiotherap* or "radiation therap*" or "radiation treatment")):ti,ab,kw or sbrt or sabr:ti,ab,kw (Word variations have been searched) |
| #5 | MeSH descriptor: [Radiotherapy, Intensity-Modulated] explode all trees |
| #6 | ("intensity modulated" near/3 (radiotherap* or "radiation therap*" or "radiation treatment")):ti,ab,kw or ("volumetric modulated arc" near/3 (radiotherap* or "radiation therap*" or "radiation treatment")):ti,ab,kw or LINAC:ti,ab,kw or (conformal near/3 (radiotherap* or "radiation therap*" or "radiation treatment")):ti,ab,kw (Word variations have been searched) |
| #7 | #1 or #2 or #3 or #4 or #5 or #6 |

| **WoK** | Search |
| --- | --- |
| # 7 | #5 AND #4 Refined by: PUBLICATION YEARS: ( 2018 OR 2010 OR 2017 OR 2016 OR 2015 OR 2014 OR 2013 OR 2012 OR 2011 ) |
| # 6 | #5 AND #4 |
| # 5 | TOPIC: ("economic evaluation*" OR pharmacoeconomic* or pharmaco-economic*) OR TOPIC: ((Cost* near/2 (Effective* or analysis* or Utility* or Benefit* or Minimi*))) OR TITLE: (economic*) OR TOPIC: ("willingness to pay") OR TOPIC: (((economic near/2 model*) or markov or "monte carlo method")) OR TOPIC: ((decision* NEXT (tree* or model* or analysis))) OR TOPIC: ((resource* NEXT (use* or utilisation))) OR TOPIC: (((healthcare or "health care" or "direct service" or hospital or drug*) NEXT cost*)) |
| # 4 | #3 OR #2 OR #1 |
| # 3 | TOPIC: (((stereota* near/3 (radiotherap* or "radiation therap*" or "radiation treatment")))) OR TOPIC: (sbrt OR sabr) |
| # 2 | TOPIC: (((("proton beam" or particle) near/3 (therap* or treatment)))) OR TOPIC: ((((proton* or particle) near/3 (radiotherap* or "radiation therap*" or "radiation treatment")))) |
| # 1 | TOPIC: (("intensity modulated" near/3 (radiotherap* or "radiation therap*" or "radiation treatment"))) OR TOPIC: (("volumetric modulated arc" near/3 (radiotherap* or "radiation therap*" or "radiation treatment"))) OR TOPIC: ((conformal near/3 (radiotherap* or "radiation therap*" or "radiation treatment"))) OR TOPIC: (IMRT OR VMAC OR LINAC) |

| **TUFT’s** | Search |
| --- | --- |
| 1 | proton beam |
| 2 | proton radiotherapy |
| 3 | proton radiation therapy |
| 4 | particle therapy |
| 5 | particle radiotherapy |
| 6 | particle radiation therapy |
| 7 | stereotactic |
| 8 | sbrt |
| 9 | sabr |
| 10 | intensity modulated |
| 11 | imrt |
| 12 | volume modulated arc |
| 13 | vmat |
| 14 | conformal |
